# Psychometric properties of the Memory Alteration Test (M@T) using a confirmatory factor analysis and a Rasch model

**DOI:** 10.1002/alz.095757

**Published:** 2025-01-09

**Authors:** Alicia Boluarte Carbajal, Arantxa Sánchez Boluarte, Brian Florentino Santisteban, Angel Zegarra López, Sofía Sanchez Boluarte

**Affiliations:** ^1^ Universidad Cesar Vallejo, Los Olivos, Lima Peru; ^2^ School of Public Health. University of Washington, Seattle, US, WA USA; ^3^ Universidad de Lima, Surco, Lima Peru

## Abstract

**Background:**

Background. According to the World Health Organization, dementia is one of the leading causes of disability in the elderly, imposing a significant burden on global public health. It is estimated that dementia affects over 55 million people worldwide, with 60% of them living in low‐ and middle‐income countries. Early detection of cognitive impairment is crucial for timely intervention and management. In this context, neuropsychological assessment tools play a vital role in identifying individuals at risk. The Memory Alteration Test (M@T) has emerged as a promising tool due to its high sensitivity for early detection of dementia and MCI. To build on this, our study aimed to analyze the psychometric properties of the M@T, in order to ensure its validity and reliability in clinical settings.

**Method:**

The study was conducted among 352 participants over 60 years of age, recruited from Municipal Elderly Care Centers in four socioeconomically distinct districts of Lima, Peru. The M@T captures five domains of memory: encoding, temporal orientation, semantic memory, free recall and cued recall. For this, we proposed a unidimensional model and a multidimensional model with 5 factors, using a confirmatory factor analysis and a Rasch modeling.

**Result:**

The majority of participants were women (82.9%), with a notable proportion reporting a clinical history of hypertension (33.2%). The internal structure analysis and Rasch modeling exhibited a unidimensional structure with good fit and adequate reliability (GY = .897, ord = .934). On the other hand, the 5‐factor multidimensional model showed a poor fit with unreliable scores (GY = .174‐.785, ord = .576‐.898). Likewise, the Rasch analysis demonstrated that the test measures a general memory ability reliably (Rp = .813), while the multidimensional model showed that the scores in each domain were not consistent (Rp = .358‐.707).

**Conclusion:**

The findings suggest that the M@T operates as a unidimensional factor, emphasizing the importance of considering the total score rather than individual dimensions. This underscores the need for caution when interpreting results, particularly when employing multidimensional models, which may not accurately capture the underlying structure of the test. Ultimately, a unidimensional approach offers a more parsimonious and reliable means of assessing cognitive function using the M@T.

